# Painless Extraction of Teeth

**Published:** 1871-11

**Authors:** 


*Painless Extraction of Teeth.*—Dr. A. C. Castle in the "Dental Cosmos," states that he has for thirty years adopted the plan of "obtunding or benumbing the extremities of the temporal nerves for painless extraction of teeth from their sockets with complete success, never having used or countenanced the exhibition of chloroform, ether, or nitrous oxide gas for this *minor* surgical operation. The benumbing, or *mechanical anæsthesia*, of the temporal branches of nerves obtunds the whole nerve to a sufficient extent to allow the teeth to be removed with sensation so slight, which, if not attending a special surgical operation, would scarcely be noticed by the patient. One of two modes may be adopted. Application of ice to the temples, which is somewhat distressing, the sensation of cold striking deeply. The other, to which I give the preference, is done by an assistant, with each of his middle fingers pressing their points (of the fingers) with persistent firmness and force into the *fossa* or hollow behind the the ridge of the temporal bone, which forms the external bone circle orbit of the eye. Pressure for one minute is all that is necessary. The practice is as simple as it is harmless, and leaves no after unpleasant sensation to annoy the patient. It is an instinctive method often adopted by people themselves, who press their temples with their fingers to relieve themselves temporarily of the acute paroxysms of nervous headache. This temporary pressure, with sufficient force, is all that is required to remove teeth painlessly."

